# Contextualizing the ASPE standards of best practice: reflecting on the past, present, and future of SP methodology

**DOI:** 10.1186/s41077-026-00480-5

**Published:** 2026-08-12

**Authors:** Amelia M. Wallace, Cory Krebsbach, Janice Radway, Lou Clark

**Affiliations:** 1 Sentara Center for Healthcare Simulation and Immersive Learning, Macon & Joan Brock Virginia Health Sciences at Old Dominion University, Norfolk, USA; 2 Department of Healthcare Simulation, Rosalind Franklin University of Medicine and Science, Chicago, USA; 3 Perelman School of Medicine, University of Pennsylvania, Philadelphia, USA; 4 Medical School, University of Minnesota, Minneapolis, USA

## Introduction

As we celebrate the publication of the Association of SP Educators Standards of Best Practice (ASPE SOBP) for Physical Exam Teaching Associates (PETAs) and corresponding literature review, we, elected leaders of ASPE, offer reflections on SP methodology history, current practices, and future opportunities. Our aim is to contextualize this addition to what is now the third SOBP publication [1, 2] that guides the global simulation community on best practices of simulated participant (SP) methodology.

Thank you to the ASPE members and SP Educators who made time to share their expertise as authors on these new standards—in addition to their daily work of actively recruiting, hiring, coaching, and safeguarding PETAs. The PETA standards and companion paper outline the global standards of practice for individuals who work with PETAs. PETAs are individuals who engage learners while teaching with their own body as the model for instruction. It is important to note that as with all of SP methodology, there can be great variation in the application of this methodology. Readers will notice the consistent framework associated with safety and inclusion of all collaborators in the design and implementation of work with PETAs. This is because the added human component of an individual (SP/GTA/MUTA/PETA) who is not a clinician or learner engaging in the educational process requires an intentional commitment to stewardship of that perspective. SP Educators are integral to this process because they are the professionals entrusted with safeguarding and advocating for PETAs while they focus on directly teaching learners. The PETA standards echo this in the domains related to safety, development of sessions, training and program management.

### Values and principles underpinning SP methodology

Central themes in all three SOBP publications are inclusivity and psychological and physical safety for all participants, including SPs [3–7]. Inclusivity means engaging SPs in sharing their perspective wherever possible (e.g. design, feedback, etc.). SP methodology asserts that the person who should have the most agency in articulating their perspective is the person who experienced the stimulus. To appreciate the current landscape and imagine future opportunities, understanding the history of SP methodology is important in order to contextualize the significance of the three ASPE SOBP publications.

### History of SP methodology

SP methodology began in the 1950 s with the innovation credited to physicians Howard Barrows, MD and Paula Stillman, MD. However, there is a largely unacknowledged and hidden group of interdisciplinary professionals, now known as SP Educators, who worked alongside Barrows and Stillman to advance SP methodology from the beginning. Their vision and resilience catalyzed the profession of SP education into existence throughout the 1980 s and 1990 s, leading to the formation of the Association of SP Educators (ASPE).

As the 1990 s and 2000 s continued, the concept of people without clinical backgrounds engaging in the educational process was galvanized by the adoption of SP methodology as an element of national licensing examinations for learners in Canada and the USA. While many medical educators and SP Educators in North America focused on summative assessment, applications of SP methodology internationally were wider ranging. This resulted in a variety of applications and an extensive lexicon of terms. As the profession expanded, so did the terms used to describe individuals associated with human simulation, including *role-players*, *patient instructors*, *programmed patients*, *teaching associates*, *simulated patients*, *standardized patients*, *simulated participants*, *patient teachers*, and *patient educators*.

In 2017, the authors of the ASPE SOBP, led by ASPE past president Karen Lewis, PhD, finalized the first iteration of the standards. The Advances in Simulation Journal, led by Debra Nestel, AM, PhD, FAcadMEd, FSSH as editor-in-chief, supported and published the SOBP [1].

### Standards and scholarship as SP methodology matures

The ASPE SOBP [1], the GTA/MUTA standards [2], and the PETA standards were formed by ASPE Standards of Practice committee members, who represent SP Educators from across the globe. The authors designed the standards to provide guidance yet remain flexible for those practicing SP methodology in a variety of contexts globally.

At present, the ASPE SOBP is widely cited and serves as the foundation for related advances in SP methodology. It has been translated into multiple languages and is undergoing its first major revision, with an anticipated publication date of 2026. The reliance on the SOBP in the literature inspired ASPE members, led by ASPE past president Gayle Gliva-McConvey, to develop the book *Implementing Best Practices of Standardized Patient Methodology,*published in 2020 as part of Springer’s Comprehensive Healthcare Simulation series [8]. Each chapter is framed by the five ASPE SOBP domains: safety, case development, training, program management, and professional development; and is undergirded by the five ASPE SOBP values: safety, quality, professionalism, accountability, and collaboration. This edited volume, featuring contributions from ASPE members around the world, serves as a “how to” manual for simulation professionals who work with SPs.

Building on this work, in 2024, a team of ten international leaders in SP methodology and research, led by ASPE immediate past president Lou Clark, PhD, MFA, published the landmark editorial “Call to action: Honoring simulated participants and collaborating with simulated participant educators” [9]. This piece provides guidance for humanistic engagement with SPs by inviting the global simulation community to adopt and advance the linguistic change of not “using” SPs, which dehumanizes them, but rather “working with and partnering with” SPs. The article also calls for acknowledgement of SP Educators’ hidden work, which contributes to SP participation in health professions education. Specifically, this publication calls for SP Educators who are ASPE members to be included on industry editorial boards, as co-authors on publications, and as members of research teams.

Finally, the ASPE SOBP serves as the foundation for ASPE Accreditation in Human Simulation. This undertaking was the first of its kind to map the ASPE SOBP domains and values to accredit SP programs. Led by ASPE past president Shawn Galin, PhD, this initiative is being adopted by programs around the world.

### Future directions/contemporary issues

The development of standards of practice for SP methodology has always followed innovation. As with the first SOBP publication, in many ways SP methodology is at another inflection point. Future considerations include partnership and integration with technology that maintains prioritizing the voice of the human patient.

For instance, artificial intelligence (AI) may help identify whether the learner has palpated the patient’s liver, and the PETA will have the information regarding pressure, comfort, and scope from the patient’s perspective. A future extension of the ASPE SOBPs may be to clarify those boundaries. Melding these perspectives upholds inclusivity between humans and technology in the safest possible context.

Why is the concept of human voice critical in terms of SPs’ contributions to health professions education? Because patients are people. We are human. As Human Simulation advances, SP Educators will continue to lead the way in creating and establishing best practices for SP methodology on the foundation of safety and inclusion for all. This well-established history demonstrates an openness for diverse perspectives, the ability to integrate emerging technologies, and the vision to leave space for those that have yet to be known.

## Data Availability

Not applicable.
